# Trajectory of Gastrointestinal Symptoms in Previously Hospitalized COVID-19 Survivors: The Long COVID Experience Multicenter Study

**DOI:** 10.3390/v15051134

**Published:** 2023-05-10

**Authors:** César Fernández-de-las-Peñas, Juan Torres-Macho, Carlos Guijarro, José D. Martín-Guerrero, Oscar J. Pellicer-Valero, Gustavo Plaza-Manzano

**Affiliations:** 1 Department of Physical Therapy, Occupational Therapy, Physical Medicine and Rehabilitation, Universidad Rey Juan Carlos, 28922 Madrid, Spain; 2 Department of Internal Medicine, Hospital Universitario Infanta Leonor-Virgen de la Torre, 28031 Madrid, Spain; juan.torresm@salud.madrid.org; 3 Department of Medicine, School of Medicine, Universidad Complutense de Madrid, 28040 Madrid, Spain; 4 Department of Internal Medicine, Hospital Universitario Fundación Alcorcón, 28922 Madrid, Spain; carlos.guijarro@urjc.es; 5 Department of Medicine, Universidad Rey Juan Carlos (URJC), 28922 Madrid, Spain; 6 Intelligent Data Analysis Laboratory, Department of Electronic Engineering, ETSE (Engineering School), Universitat de València (UV), 46010 Valencia, Spain; jose.d.martin@uv.es; 7 Image Processing Laboratory (IPL), Universitat de València, Parc Científic, Paterna, 46010 València, Spain; oscar.pellicer@uv.es; 8 Department of Radiology, Rehabilitation and Physiotherapy, Universidad Complutense de Madrid (UCM), IdISSC, 28040 Madrid, Spain; gusplaza@ucm.es

**Keywords:** COVID-19, gastrointestinal, diarrhea, symptoms, trajectory, Sankey plots

## Abstract

This multicenter cohort study used Sankey plots and exponential bar plots to visualize the fluctuating evolution and the trajectory of gastrointestinal symptoms in previously hospitalized COVID-19 survivors during the first 18 months after acute SARS-CoV-2 infection. A total of 1266 previously hospitalized COVID-19 survivors were assessed at four points: hospital admission (T0), at 8.4 months (T1), at 13.2 months (T2), and at 18.3 months (T3) after hospitalization. Participants were asked about their overall gastrointestinal symptoms and particularly diarrhea. Clinical and hospitalization data were collected from hospital medical records. The prevalence of overall gastrointestinal post-COVID symptomatology was 6.3% (n = 80) at T1, 3.99% (n = 50) at T2 and 2.39% (n = 32) at T3. The prevalence of diarrhea decreased from 10.69% (n = 135) at hospital admission (T0), to 2.55% (n = 32) at T1, to 1.04% (n = 14) at T2, and to 0.64% (n = 8) at T3. The Sankey plots revealed that just 20 (1.59%) and 4 (0.32%) patients exhibited overall gastrointestinal post-COVID symptoms or diarrhea, respectively, throughout the whole follow-up period. The recovery fitted exponential curves revealed a decreasing prevalence trend, showing that diarrhea and gastrointestinal symptoms recover during the first two or three years after COVID-19 in previously hospitalized COVID-19 survivors. The regression models did not reveal any symptoms to be associated with the presence of gastrointestinal post-COVID symptomatology or post-COVID diarrhea at hospital admission or at T1. The use of Sankey plots revealed the fluctuating evolution of gastrointestinal post-COVID symptoms during the first two years after infection. In addition, exponential bar plots revealed the decreased prevalence of gastrointestinal post-COVID symptomatology during the first three years after infection.

## 1. Introduction

The current understanding supports the idea that coronavirus disease, 2019 (COVID-19) is a multi-systematic disease that affects multiple organs, including the respiratory, cardiovascular, musculoskeletal, and gastrointestinal systems, among others [1]. This multi-organ manifestation has been related to the cytokine-associated storm that results in endothelial inflammation and microvascular thrombosis, leading to multiple organ failure [2,3].

Among the varied symptomatology (e.g., fever, dyspnea, anosmia, ageusia, throat pain, chest pain) that an individual can experience during the acute phase of severe acute respiratory syndrome coronavirus 2 (SARS-CoV-2) infection, gastrointestinal symptoms are reported by almost 60% of patients who visit the emergency room due to COVID-19 [4]. In fact, some gastrointestinal symptoms occur with or before respiratory symptoms in patients with COVID-19 [5]. The receptor for COVID-19, the angiotensin-converting enzyme-2 (ACE2), is found throughout the gastrointestinal tract, confirming the pathophysiologic relevance of gastrointestinal symptoms in SARS-CoV-2 [6]. In such a scenario, intestinal weakness may promote the progression of SARS-CoV-2 through the gut–lung axis and increase the severity of COVID-19 [7]. This hypothesis is supported by the fact that the presence of overall gastrointestinal symptoms is associated with severe COVID-19 [8]. Nevertheless, it is possible that this association is symptom-specific since diarrhea is a risk factor of severe COVID-19 whereas other symptoms, e.g., nausea, vomiting, or abdominal pain, are limited risk factors of severe COVID-19 [9,10]. Moreover, Nobel et al. found that patients presenting gastrointestinal symptoms at onset were associated with mild COVID-19 disease and lower mortality rates [11]. Accordingly, the relevance of gastrointestinal symptoms needs to be further elucidated. 

Gastrointestinal symptoms are also present after the acute phase of the infection. The presence of symptoms after an acute phase of SARS-CoV-2 infection is called long COVID [12] or post-COVID-19 condition [13]. Although there are different definitions for the presence of post-COVID symptoms, the term post-COVID-19 condition has been proposed and has been defined as follows: “Post-COVID-19 condition occurs in people with a history of probable or confirmed SARS-CoV-2 infection, usually three months from the onset of COVID-19 with symptoms that last for at least two months and cannot be explained by an alternative diagnosis” [13]. More than 100 post-COVID symptoms have been described, with fatigue, dyspnea, brain fog, and pain being the most prevalent [14]. Several studies have found that the prevalence rates of gastrointestinal post-COVID symptoms range from 10% to 25% the first 6 months after infection [15,16,17]. The meta-analysis conducted by Choudhury et al. found an overall prevalence of 22% for gastrointestinal symptoms in patients with long COVID (n = 158,731 subjects) [18]. In fact, gastrointestinal symptoms are rated as the most difficult post-COVID symptom in 11% of patients [19]. Accordingly, better understanding of the long-lasting prevalence of gastrointestinal post-COVID symptomatology is clearly needed. 

There is evidence showing that the gut microbiome’s composition is altered in patients with post-COVID symptoms; hence, a putative underlying mechanism associating long COVID with gastrointestinal symptomatology is gut dysbiosis [20,21]. In fact, one of the hypotheses explaining long COVID is related to the persistence of SARS-CoV-2 (viral persistence) in the gut microbiota [22]. The pathological symptoms raise the possibility that the gastrointestinal tract acts as a secondary reservoir site for SARS-CoV-2 tropism and infection [23]. Natajaran et al. [24] found fecal SARS-CoV-2 RNA in 49.2% of COVID-19 survivors the first week after diagnosis, in 12.7% of individuals at 4 months after diagnosis, and in 3.8% of individuals at 7 months after diagnosis. Furthermore, gastrointestinal symptoms were found to be associated with the fecal shedding of SARS-CoV-2 RNA. Accordingly, understanding the trajectory of these gastrointestinal symptoms could have significant implications for the diagnosis, triaging, and management of individuals with long COVID.

Most published studies investigating the presence of post-COVID gastrointestinal symptoms have used cross-sectional study designs to conduct just one assessment of the presence of these post-COVID symptoms and have follow-up periods shorter than one year [18]. A previous study (the Long COVID Experience) analyzed the recovery curve of gastrointestinal symptoms from the acute phase of the infection up to a year after hospitalization in a cohort of previously hospitalized COVID-19 survivors [25]. Here, we present a follow-up analysis of the Long COVID Experience study by including data from the onset of the acute infection up to 18 months after hospital discharge. In addition, we used Sankey plots as a new method for visualizing the fluctuating evolution of gastrointestinal symptomatology during the first 18 months after acute SARS-CoV-2 infection. Sankey plots are flow diagrams that visualize the evolution of a specific symptom over time by representing snapshots of the status of each individual at particular time points (see Section 2.3). Finally, we used an exponential bar plot to analyze the exponential trajectory of gastrointestinal symptomatology after infection during a follow-up period longer than one year. An exponential bar plot is a curve that has an increasing or decreasing slope based on the available prevalence data (see Section 2.4).

## 2. Methods

### 2.1. Participants

The Long COVID Experience is a multicenter cohort study. Its subjects comprise previously hospitalized COVID-19 survivors whose diagnosis of SARS-CoV-2 infection (ICD-10 code) was confirmed by a real-time reverse transcription-polymerase chain reaction (RT-PCR) assay of nasopharyngeal/oral swab samples at hospital admission during the first wave of the pandemic (from 10 March to 31 May 2020) in five hospitals (Hospital Universitario Severo Ochoa, Hospital Universitario Fundación Alcorcón, Hospital Universitario Infanta Leonor, Hospital Universitario de Fuenlabrada, and Hospital Universitario Clínico San Carlos) in Madrid (Spain). As previously described, from among all patients hospitalized during the first wave of the pandemic (n = 7150), a sample of 400 individuals from each hospital was randomly selected using online software. The ethics committees of all the hospitals involved approved the study (HUFA20/126, HUIL/092-20, HCSC20/495E, HSO25112020, HUF/EC1517). Verbal informed consent was obtained from all participants before any data were collected. 

### 2.2. Procedure

The procedure of this multicenter cohort study was described previously [25]. Briefly, clinical and hospitalization data were collected from hospital medical records. We recorded symptoms experienced at the onset of infection, days at hospital, previous medical co-morbidities, and internal care unit admission. In addition, participants were scheduled for semi-structured telephone interviews, which were conducted by trained healthcare professionals, at six, twelve, and eighteen months after hospitalization. Participants were systematically asked about the presence of overall gastrointestinal symptoms (i.e., abdominal pain, nausea, or vomiting) that they attributed to the SARS-CoV-2 infection. We separated these overall gastrointestinal symptoms from diarrhea since the presence of diarrhea during the acute phase of SARS-CoV-2 infection has been associated with poor hospitalization outcomes and COVID-19 severity [26,27].

### 2.3. Sankey Plots

Sankey plots are flow diagrams used to visualize the flow of quantitative data; they allow for the assessment of the evolution of patients over time [28]. The X axis represents the time points (in the current study, at onset, and six, twelve, and eighteen months after), while the Y axis represents the percentage of individuals suffering (or not) from a particular symptom (overall gastrointestinal symptoms or diarrhea). The darker vertical bars are the nodes of the Sankey diagram, which represent a snapshot of the state of the individuals at that particular time point. The arcs depict the flows of patients between the states, with a width that is proportional to the percentage (from the total sample) of participants in that flow. The percentage of individuals with/without the symptom is annotated on the right side of the nodes, whereas the flows themselves, with the percentage of individuals that they contain, are annotated on the left side of the nodes [28].

### 2.4. Exponential Bar Plots

The exponential curves were created using Matplotlib 3.3.4 and were fitted to the following formula: *y* = *Ke^ct^*, where *y* represents the modeled prevalence of the symptom (overall gastrointestinal symptomatology or diarrhea) at a time *t* (in months), and *K* and *c* are the parameters of the model. 

### 2.5. Statistical Analysis

Finally, using Python’s library statsmodels 0.11.1, multivariate logistic regressions were performed to identify variables collected at hospital admission (COVID-19 onset and follow-up) associated with the development of gastrointestinal symptoms at twelve and eighteen months after hospital discharge. Adjusted odds ratios (ORs) with their confidence intervals (95% CI) are presented. A priori, the level of significance was set at 0.05.

## 3. Results

From a total of 2000 individuals who were randomly selected and invited to participate, 1969 (46.5% women, age: 61, SD: 16 years) were included at baseline (T0) and 6 months (T1), 1593 were assessed at 12 months (T2), and 1266 were evaluated at 18 months (T3). Accordingly, analyses were fitted based on the sample that completed all assessments (n = 1266, 64.3%). Participants were assessed at T1 (mean: 8.4, range 6 to 10), T2 (mean: 13.2, range 11 to 15), and T3 (mean: 18.3, range 16 to 21) months after hospital discharge. Table 1 shows the clinical and hospitalization data of the sample patients. 

**Figure 1:**
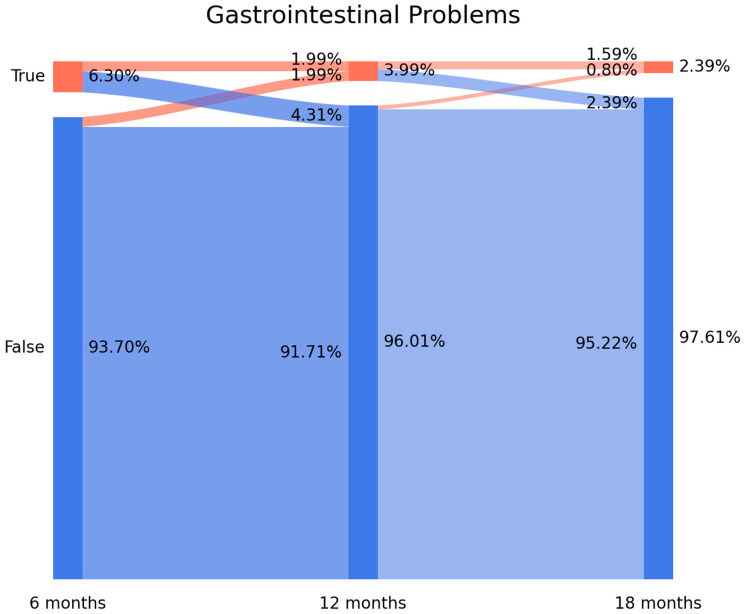
Sankey plots of overall gastrointestinal symptoms at (from left to right) T1 (8.4 months after hospital discharge) vs. T2 (13.2 months after hospital discharge) and vs. T3 (18.3 months after hospital discharge). The X axis represents the time points while the Y axis represents the percentage of individuals suffering (or not suffering) from gastrointestinal symptomatology. The darker vertical bars represent the percentage of individuals who, at that particular time point, were negative or positive for overall gastrointestinal symptomatology.

The prevalence of overall gastrointestinal post-COVID symptomatology was 6.3% (n = 80) at T1, 3.99% (n = 50) at T2, and 2.39% (n = 32) at T3. Figure 1 shows the Sankey plots of overall gastrointestinal post-COVID symptomatology. Figure 1 shows that 68.75% of individuals (n = 55/80) experiencing gastrointestinal post-COVID symptoms at T1 did not report symptomatology at T2 (4.31% arc from true at T1 to false at T2). Interestingly, 1.99% (n = 25) of individuals who did not report gastrointestinal symptoms at T1 reported some symptomatology at T2. Finally, 60% (n = 30/50) of subjects with symptoms at T2 did not report them at T3 (2.39% arc from true at T2 to false at T3). The Sankey plot revealed that just 20 patients (1.59% of the sample) exhibited overall gastrointestinal post-COVID symptomatology throughout the whole follow-up period. 

**Figure 2:**
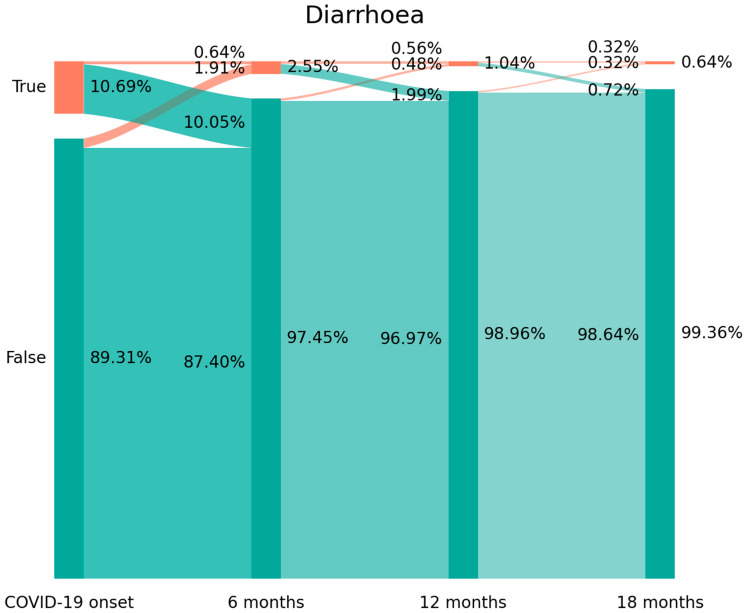
Sankey plots of diarrhea at (from left to right) T0 (hospital admission, COVID-19 onset) vs. T1 (8.4 months after hospital discharge), vs. T2 (13.2 months after hospital discharge), and vs. T3 (18.3 months after hospital discharge). The X axis represents the time points while the Y axis represents the percentage of individuals suffering (or not suffering) from diarrhea. The darker vertical bars represent the percentage of individuals who, at that particular time point, are negative or positive for diarrhea.

The prevalence of diarrhea decreased from 10.69% (n = 135) at hospital admission (T0), to 2.55% (n = 32) at T1, to 1.04% (n = 14) at T2, and to 0.64% (n = 8) at T3. Figure 2 shows the Sankey plots for diarrhea, and indicates that 94% of those individuals (n = 127/135) experiencing diarrhea at hospital admission (T0) had recovered at T1 (10.05% arc from true at T0 to false at T1). In fact, 75% (n = 24/32) of individuals reporting diarrhea at T1 had developed “de novo” post-COVID symptoms, since they did not experience diarrhea at T0 (1.91% arc from false at T0 to true at T1). A similar tendency was observed between T1 and T2 and T2 and T3. The Sankey plot reveals that just 4 patients (0.32% of the sample) suffered from diarrhea from the onset of the infection and throughout the whole follow-up period.

**Figure 3:**
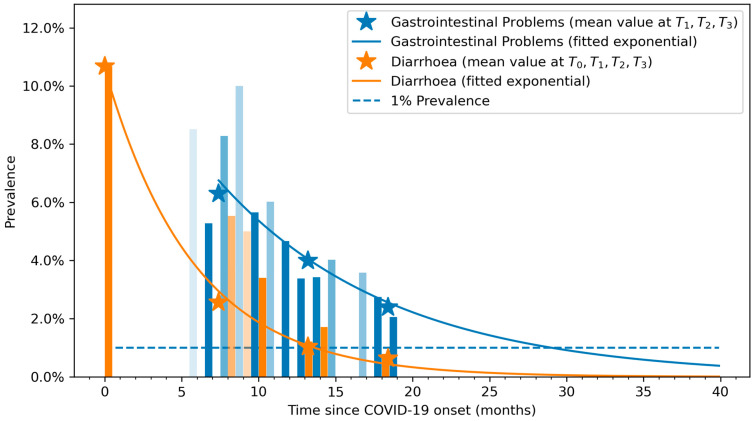
Exponential recovery curve of self-reported diarrhea (in orange) and overall gastrointestinal (in light blue) symptoms. Opacity approximately indicates the sample size at that follow-up time. Asterisks represent the mean values at the T0, T1, T2, and T3 follow-up points.

Figure 3 graphs the fitted exponential curves, showing decreased prevalence trends in both overall gastrointestinal symptoms and diarrhea. The vertical bars represent the percentage of patients that have diarrhea (in orange) or gastrointestinal symptoms (blue) at any given time (opacity indicates the sample size at that time). The mean prevalence values at each follow-up period (T0, T1, T2, and T3) are marked with asterisks in the graphs. 

The regression models did not reveal any potential symptoms at hospital admission or at T1 (eight months after infection) to be associated with the presence of gastrointestinal post-COVID symptomatology or post-COVID diarrhea at 12 (Table 2) or 18 (Table 3) months. 

## 4. Discussion

As far as the authors know, this is the first study to use two different visualization approaches (i.e., Sankey plots and exponential curves) to analyze the recovery of post-COVID gastrointestinal symptoms in a cohort of previously hospitalized COVID-19 survivors. The use of Sankey plots revealed the fluctuating evolution of gastrointestinal post-COVID symptoms during the first years after infection. In addition, exponential bar plots revealed a progressive decrease in the prevalence of gastrointestinal post-COVID symptoms during the first years after infection

We analyzed overall gastrointestinal symptoms and diarrhea separately. Previous cross-sectional studies reported an overall prevalence of gastrointestinal post-COVID symptoms of around 20% during the first 6 months after infection [15,16,17]. Our study found prevalence rates of gastrointestinal post-COVID symptoms ranging from 6.3% to 2.4%, figures lower than those presented in the available literature [15,16,17,18]. Differences in study designs (cross-sectional vs. longitudinal) and shorter follow-up periods (one and six months after infection) could explain the low prevalence rates found in our study in comparison with previous studies. In fact, the exponential recovery curves indicate that the prevalence of gastrointestinal post-COVID symptomatology decreased with time; therefore, longer follow-up periods would lead to lower prevalence rates. It is important to note that gastrointestinal symptomatology was not assessed at hospital admission, since most patients reported only diarrhea or vomiting. It is possible that overall gastrointestinal symptoms may be underreported during the acute phase of SARS-CoV-2 infection if not specifically investigated, since they are not as bothersome as other COVID-19 onset symptoms such as fever, dyspnea, or chest pain. 

Diarrhea is the only symptom experienced by some patients during the acute phase of the infection and is present in approximately 10–20% of patients [29]. The prevalence rate we found for diarrhea at hospital admission (10.7%) agrees with the currently available data [29]. Similarly, the prevalence of diarrhea as a post-COVID symptom has been found to be around 10% during the first few months after the infection [18]. Our prevalence rates for post-COVID diarrhea (0.64–2.5%) were also lower than those suggested by previous data [18]. Again, the exponential recovery curves indicate that the prevalence of post-COVID diarrhea decreased with time; hence, longer follow-up periods would lead to lower prevalence rates.

It has been proposed that the presence of gastrointestinal post-COVID symptoms can lead to a diagnosis of post-infection functional gastrointestinal disorders (FGIDs) [30,31]. Others proposed the term post-COVID-19 Disorders of Gut–Brain Interaction (DGBI) to describe this condition [8]. One of the most interesting findings of the current analysis is that Sankey plots allow us to identify the fluctuating nature of gastrointestinal post-COVID symptoms. In fact, it was previously suggested that post-COVID-19 has a fluctuating nature [32]. However, data supporting this assumption are still scarce. Burton et al. recently reported that long COVID symptomatology varies in each individual over short time periods [33]. Furthermore, the concepts of a “new-onset post-COVID symptom” or a “persistent symptom” have also been proposed [34] but not investigated in epidemiological studies. Analyzing the Sankey plots, the current results identified the presence of:

1. New-onset symptoms: subjects experiencing a symptom, e.g., diarrhea, which they did not report during the acute phase of infection (1.91% arc from false at T0 to true at T1 on Figure 2).
2. Delayed-onset symptoms: individuals reporting a symptom at a longer follow-up period after the acute phase of the infection (0.48% arc from false at T1 to true at T2 at Figure 2 or 1.99% from false at T1 to true at T2 at Figure 1).
3. Persistent symptoms: a small proportion of patients (0.32% of the sample for diarrhea, shown in Figure 2, or 1.59% of the sample for overall gastrointestinal symptoms, shown in Figure 1) exhibit a symptom from the beginning of the acute infection up to 18 months afterwards.

Both new-onset and persistent post-COVID symptoms are easily attributable to COVID-19. The presence of a new-onset post-COVID symptom is considered in the definition of post-COVID-19 condition if the symptom started during the first three months after the acute infection: “…symptoms attributed to SARS-CoV-2 infection should appear no later than two months from the onset of the disease [13]…”. All patients experiencing the presence of overall gastrointestinal symptomatology and/or diarrhea at T1 reported that the symptoms started soon after the acute infection. Surprisingly, the term persistent post-COVID symptom is not addressed in or integrated into the definition of post-COVID-19 condition [13]; however, this is the situation in which a symptom can most easily be attributed to a SARS-CoV-2 infection since the symptom appeared during the acute phase and did not disappear afterwards. In fact, the presence of gastrointestinal symptoms during the acute phase of the infection is associated with gastrointestinal post-COVID symptoms six months after the acute infection [35], supporting the presence of a persistent symptom; however, these results were not replicated in the current study at later follow-up points. The term “delayed-onset symptom” is also not considered in the definition of post-COVID-19 condition [13]; indeed, it is difficult to conclusively attribute this kind of post-COVID symptom, which appears months after the infection, to SARS-CoV-2 because of its delayed appearance in relation to the end of the onset phase. In line with this hypothesis, Blackett et al. observed that mental health conditions were associated with the presence of gastrointestinal post-COVID symptomatology, suggesting that gastrointestinal consequences may also be partially associated with heightened stress and anxiety arising from the contextual aspects of COVID-19 [16]. Similar results were identified by Vélez et al., who also noted that depression and anxiety were associated with the development of gastrointestinal post-COVID symptomatology [36]. In addition, it is plausible that the development of gastrointestinal symptomatology one year after SARS-CoV-2 infection may be related to other factors different than COVID-19, such as the development of medical comorbidities. 

The use of an exponential recovery curve suggests that gastrointestinal symptomatology can last up to three to four years after infection, but the natural tendency of these symptoms is to disappear. In fact, most patients did not report the use of any medication for specifically treating these symptoms; accordingly, it is to be expected that the resolution and the decreasing tendency observed in the recovery curve represents “the natural evolution of these symptoms”. Nevertheless, since gastrointestinal cells exhibit high expression of the ACE2 receptor, the SARS-CoV-2 virus can lead to gastrointestinal inflammation [37]. A long-lasting inflammatory state would support the hypothesis that the gastrointestinal tract acts as a secondary reservoir site (i.e., the viral persistence theory) for SARS-CoV-2 tropism and infection [23]. Therefore, since the presence of gastrointestinal symptoms can persist for years, these symptoms may be responsible for enhanced SARS-CoV-2 exposure and the presence of significant viral loads for years in these patients. Identifying the potential risk factors associated with the development of long-lasting gastrointestinal symptoms could enable the more effective management of people with long COVID. Unfortunately, we were not able to find any factors associated with overall gastrointestinal post-COVID symptoms or with post-COVID diarrhea, probably because of the long-term follow-up periods included in our study. 

Although this study used two novel methods to visualize and analyze post-COVID symptoms, its potential weaknesses are recognized. First, the study only included a cohort of COVID-19 survivors who were hospitalized during the first wave of the outbreak and, accordingly, were originally infected with the historical strain. Additionally, our sample included individuals with a mean age of 61 years with different medical co-morbidities (see Table 1); as such, the results presented here should be considered in the context of this population of COVID-19 survivors. Second, we only investigated gastrointestinal symptoms. Interactions between the different post-COVID symptoms are common. Third, we collected data by telephone interviews, a procedure that could involve potential bias. Nevertheless, it is important to note that the use of telephone interviews is the only way to assess large cohorts like that assessed in the current study (over 1000 patients across long-term follow-up periods). Fourth, the presence of gastrointestinal symptoms was self-reported, and the informed diagnosis of FGIDs or DGBI requires the use of valid instruments, e.g., a valid Rome IV Adult Questionnaire [38]. Finally, we cannot assume that the development of symptoms one year after infection (delayed-onset symptoms) is due to the initial infection. In fact, we did not collect data about reinfections or the patients’ vaccination status, which could explain how the presence of this symptomatology is potentially not related to the original infection and may affect the results. However, this is unlikely, since the effect of COVID-19 vaccines on people with ongoing long COVID symptoms is heterogeneous [39] and the prevalence rates observed in our study were relatively small.

## 5. Conclusions

The use of Sankey plots revealed the fluctuating evolution of gastrointestinal post-COVID symptoms during the first two years after infection in a cohort of individuals who had been hospitalized due to SARS-CoV-2 infection. In addition, exponential bar plots visualized a decrease in gastrointestinal post-COVID symptoms, including diarrhea, during the first three years after the acute infection.

## Figures and Tables

**Table 1:** Demographic and clinical data of the sample patients (n = 1266).

Age, mean (SD), years	61 (16.5)
Female (%)	578 (45.6%)
Weight, mean (SD), kg.	74.5 (14.5)
Height, mean (SD), cm.	165 (19.0)
Main symptoms at hospital admission, n (%)—T0	
Fever	948 (74.9%)
Dyspnoea	361 (28.5%)
Myalgia	374 (29.5%)
Cough	360 (28.4%)
Headache	135 (16.7%)
Diarrhea	105 (8.3%)
Anosmia	105 (8.3%)
Ageusia	66 (7.0%)
Throat pain	66 (5.2%)
Vomiting	39 (3.0%)
Medical co-morbidities	
Hypertension	336 (26.5%)
Diabetes	158 (12.5%)
Cardiovascular disease	141 (11.2%)
Asthma	85 (6.7%)
Obesity	57 (4.5%)
Chronic obstructive pulmonary disease	47 (3.7%)
Rheumatological disease	16 (1.3%)
Other (cancer, kidney disease)	207 (16.3%)
Stay at the hospital, mean (SD), days	10.5 (10.8)
Intensive care unit (ICU) admission	78 (6.2%)

**Table 2:** Adjusted odds ratio (95% confidence interval) of the multivariate regression analyses at the T2 follow-up period (13.2 months).

	Gastrointestinal Symptoms	Diarrhea
Age	0.994 (0.964; 1.025)	0.968 (0.921; 1.017)
Female sex	0.441 (0.169; 1.147)	1.239 (0.254; 6.050)
Weight	0.976 (0.942; 1.011)	0.982 (0.930; 1.037)
Height	0.982 (0.243; 3.963)	0.955 (0.943; 1.050)
Symptoms at hospital admission—T0		
Dyspnoea	1.263 (0.519; 3.073)	0.680 (0.128; 3.613)
Myalgia	1.165 (0.508; 2.674)	0.246 (0.033; 1.838)
Cough	1.121 (0.464; 2.710)	0.646 (0.122; 3.426)
Headache	0.864 (0.315; 2.370)	0.341 (0.038; 3.071)
Diarrhea	1.462 (0.449; 4.760)	0.689 (0.084; 5.626)
Anosmia	0.414 (0.132; 1.293)	1.617 (0.128; 20.382)
Ageusia	0.356 (0.055; 2.290)	0.620 (0.025; 15.142)
Throat pain	1.938 (0.467; 8.033)	1.799 (0.214; 15.130)
Vomiting	0.396 (0.040; 3.957)	0.929 (0.164; 5.246)
Medical co-morbidities		
Hypertension	1.194 (0.048; 2.920)	1.864 (0.330; 10.538)
Diabetes	0.400 (0.099; 1.620)	0.993 (0.120; 8.218)
Cardiovascular disease	0.991 (0.316; 3.112)	1.023 (0.983; 1.065)
Asthma	0.453 (0.083; 2.485)	4.006 (0.466; 34.423)
Obesity	0.855 (0.100; 7.293)	0.671 (0.192; 2.340)
Chronic obstructive pulmonary disease	0.561 (0.055; 5.677)	0.868 (0.180; 4.194)
Rheumatological disease	0.361 (0.015; 8.858)	1.373 (0.717; 3.724)
Stay at the hospital	1.002 (0.966; 1.041)	0.971 (0.866; 1.088)

**Table 3:** Adjusted odds ratio (95% confidence interval) of the multivariate regression analyses at the T3 follow-up period (18.3 months).

	Gastrointestinal Symptoms	Diarrhea
Age	1.000 (0.962; 1.039)	0.995 (0.959; 1.032)
Female sex	0.817 (0.279; 2.392)	2.495 (0.798; 7.803)
Weight	0.981 (0.942; 1.022)	1.004 (0.971; 1.038)
Height	0.998 (0.985; 1.012)	0.984 (0.967; 1.001)
Symptoms at hospital admission—T0		
Dyspnoea	0.565 (0.173; 1.849)	2.056 (0.784; 5.387)
Myalgia	1.801 (0.663; 4.891)	0.596 (0.207; 1.717)
Cough	1.689 (0.621; 4.594)	0.844 (0.286; 2.485)
Headache	0.308 (0.066; 1.440)	2.427 (0.807; 7.298)
Diarrhea	0.942 (0.227; 3.916)	1.498 (0.435; 5.154)
Anosmia	0.307 (0.032; 2.958)	0.878 (0.147; 5.230)
Ageusia	0.660 (0.128; 3.417)	1.062 (0.184; 6.134)
Throat pain	0.943 (0.127; 6.980)	0.376 (0.029; 4.965)
Vomiting	0.492 (0.036; 6.802)	4.198 (0.626; 28.150)
Medical co-morbidities		
Hypertension	1.735 (0.570; 5.281)	1.305 (0.478; 3.562)
Diabetes	0.614 (0.114; 3.302)	1.619 (0.447; 5.868)
Cardiovascular disease	1.145 (0.271; 4.842)	2.973 (0.923; 9.579)
Asthma	1.888 (0.434; 8.223)	0.797 (0.165; 3.850)
Obesity	1.055 (0.160; 6.962)	2.091 (0.268; 16.294)
Chronic obstructive pulmonary disease	3.070 (0.273; 34.578)	2.300 (0.452; 11.708)
Rheumatological disease	0.361 (0.015; 8.858)	0.441 (0.041; 4.765)
Stay at the hospital	0.965 (0.909; 1.025)	0.983 (0.938; 1.030)

## Data Availability

All data derived are included in the text.
